# PCR-Based Techniques for Leprosy Diagnosis: From the Laboratory to the Clinic

**DOI:** 10.1371/journal.pntd.0002655

**Published:** 2014-04-10

**Authors:** Alejandra Nóbrega Martinez, Carolina Talhari, Milton Ozório Moraes, Sinésio Talhari

**Affiliations:** 1 Laboratório de Hanseníase, Instituto Oswaldo Cruz - Fiocruz, Rio de Janeiro, Rio de Janeiro, Brazil; 2 Fundação Alfredo da Matta, Manaus, Amazonas, Brazil; 3 Universidade Nilton Lins, Manaus, Amazonas, Brazil; Emory University, United States of America

## Abstract

In leprosy, classic diagnostic tools based on bacillary counts and histopathology have been facing hurdles, especially in distinguishing latent infection from active disease and diagnosing paucibacillary clinical forms. Serological tests and IFN-gamma releasing assays (IGRA) that employ humoral and cellular immune parameters, respectively, are also being used, but recent results indicate that quantitative PCR (qPCR) is a key technique due to its higher sensitivity and specificity. In fact, advances concerning the structure and function of the *Mycobacterium leprae* genome led to the development of specific PCR-based gene amplification assays for leprosy diagnosis and monitoring of household contacts. Also, based on the validation of point-of-care technologies for *M. tuberculosis* DNA detection, it is clear that the same advantages of rapid DNA detection could be observed in respect to leprosy. So far, PCR has proven useful in the determination of transmission routes, *M. leprae* viability, and drug resistance in leprosy. However, PCR has been ascertained to be especially valuable in diagnosing difficult cases like pure neural leprosy (PNL), paucibacillary (PB), and patients with atypical clinical presentation and histopathological features compatible with leprosy. Also, the detection of *M. leprae* DNA in different samples of the household contacts of leprosy patients is very promising. Although a positive PCR result is not sufficient to establish a causal relationship with disease outcome, quantitation provided by qPCR is clearly capable of indicating increased risk of developing the disease and could alert clinicians to follow these contacts more closely or even define rules for chemoprophylaxis.

## Introduction

Leprosy is a chronic infectious disease caused by *M. leprae*, a slow-growing intracellular mycobacteria with tropism for Schwann cell in nerves and macrophages in the skin. In some patients, the disease is challenging to diagnose since there is no gold-standard method to differentiate between infection and disease. Leprosy is also a neglected disease, being endemic in developing countries, where detection rates show only a slight trend toward a decrease in disease (or number of cases) in spite of good treatment and the efforts of the World Health Organization (WHO) to improve the quality of leprosy control programs [1]. It is accepted that transmission occurs from human to human through the upper airways, although intermediate hosts like armadillos may play a role in certain places, such as the United States [2]. It is generally held that untreated multibacillary (MB) patients are the most important source of transmission, which occurs when bacilli are spread—usually by airborne droplets from nasal and/or mouth. Hence, leprosy patients, especially those with high bacterial load, release billions of bacilli that can potentially contaminate their close relatives or household contacts. As a result, the contacts of leprosy patients are known to have a higher risk of illness than the general population. Surveillance of these contacts would be an easy control strategy to block transmission, as suggested by the World Health Organization.

However, the steady number of new cases of leprosy in endemic countries is thought to result from the perpetuating reservoir of *M. leprae*-infected contacts and/or from the difficulties of early clinical diagnosis. It has been shown that good surveillance of patients' contacts has increased the detection rate of less severe clinical presentations with lower bacteriological indices [3], [4].

Immunological tools to detect *M. leprae* are based on their ability to detect major unique components like phenolic glycolipid-I (PGL-I), specific proteins by means of monoclonal and polyclonal antibodies [5], [6], or T cell immune response as measured by IFNγ production [7], [8]. Notwithstanding, the development of good diagnostic tests for leprosy is halted by the diversity of the strength of the cellular and humoral responses, varying from high to low (non)responders. On one hand, a major difficulty concerns paucibacillary (PB) forms, in which bacilli or antibodies against it are not easily detected in most cases. These PB patients exhibit cell-mediated immunity, secreting high levels of IFNγ after in vitro stimulation with specific *M. leprae* antigens (or a peptide fraction). On the other hand, MB patients do not produce IFNγ in vitro but have high bacillary loads that are easily identified by PCR or anti-PGL-I detection. Concerning IFNγ release, one problem in early diagnosis is that most of the household contacts show a similar pattern of IFNγ secretion as that of PB patients [9]. Generally, contacts exhibit a sustained high production of IFNγ that is dependent on continuous exposure to an infective source, i.e., a MB or sometimes a PB patient.

Both serological and immunological tests have limitations, and neither one can be considered a reliable diagnostic tool. Nevertheless, it is known that experienced clinicians and well-equipped clinics with histopathological examinations and bacillary counts, along with other clinical tests, available can diagnose most of the cases. However, the lack of a gold standard test for leprosy and the inability to distinguish infected individuals from those exhibiting active disease makes leprosy diagnosis essentially based on clinical features. Given that recognition of the disease is required, late diagnosis is relatively frequent in many patients. In addition, the lack of a specific and sensitive test to determine whether the infection has progressed to active disease makes it difficult to interrupt the transmission chain and impairs leprosy control.

Detection by PCR of *M. leprae* DNA in difficult-to-diagnose cases favors correct diagnosis and the possibility of early identification. In fact, the development and constant improvement of molecular tests for leprosy diagnosis has revealed that clinical manifestations like pure neural leprosy (PNL) are much more common than originally thought [10]–[12]. Here, we review several studies that discuss PCR usefulness in the clinical practice, such as in indeterminate leprosy, with patients who have clinical signs of leprosy but no confirmation through routine tests and histopathology, in difficult-to-diagnose cases, and in early detection in household contacts (Box 1).

**Box 1. There Is a Future for PCR in Leprosy Diagnosis**

- Surveillance of household contacts of leprosy patients favors early diagnosis of the disease.
- Semiautomatic, large-scale, cost-affordable quantitative PCR (qPCR) could be used to screen high-risk contacts and indicate chemoprophylaxis;
- qPCR can be used to diagnose leprosy in difficult-to-diagnose cases such as pure neural or atypical skin clinical presentations;
- point-of-care molecular-based technologies are available and could be used for diagnosis of leprosy, among other neglected diseases.

## Historical Aspects of Biochemical and Genetic Studies of *M. leprae*

Historically, along with the spectrum of clinical forms of leprosy, one of the problems in developing new diagnostic tests has been an inability to grow *M. leprae* in vitro. Initial studies of biochemical and molecular features of this mycobacteria species could be achieved only after the development of techniques for growing leprosy bacillus in the mouse footpad [13] and armadillos [14]. These models aided leprosy research, the development of new chemotherapeutic agents, and the confirmation of drug resistance and the antigenic and molecular structure of *M. leprae*.

The first methods to amplify *M. leprae* DNA, based on polymerase chain reaction (PCR), were developed a little longer than 20 years ago [15], [16]. Later, another wave of significant progress in understanding the molecular biology of *M. leprae* came about after the completion of the genome sequencing of the leprosy bacillus described by Cole and colleagues [17] came out, along with other mycobacterial genomes allowing comparisons [18]. Since then, bioinformatics and new-generation sequencing approaches have provided information capable of supporting studies aimed at a better understanding of *M. leprae* genetic diversity [19], [20]. In fact, it is astonishing that *M. leprae* has presented a very stable genome for a very long time. Samples recovered from skeletons are genetically conserved as compared to modern strains [21]. The information about *M. leprae* genomes also enabled isolation and characterization of genes and expression profiles. Recently, DNA microarrays shed light on *M. leprae* gene function and provided further understanding of the pathogenesis of leprosy [22]–[27]. In addition, these new technologies have proven useful in leprosy diagnosis, drug resistance detection, and for information about transmission and mycobacterial variability in high- and low-endemic areas [28]–[33]. Furthermore, a detailed review encloses information on pseudogenes, molecular epidemiology, and biology of *M. leprae* [34]; thus, these issues will not be covered here.

## PCR as a Detection Tool

In the past 20 years, definitive identification of *M. leprae* has been possible through the development of methods for the extraction, amplification, and identification of *M. leprae* DNA in clinical specimens using PCR. This technique has been applied not only to skin biopsy samples, but also to several different types of specimens such as skin smears, nerves, urine, oral or nasal swabs, blood, and ocular lesions [11], [35]–[41]. Different sequences were used as targets for PCR, such as genes encoding the 36-kDa antigen [42], 18-kDa antigen [43], 65-kDa antigen [44], complex 85 [37], 16S rDNA [45], and the repetitive sequences [46] among other *M. leprae* genes. More recently, real-time PCR technology has improved detection, increasing sensitivity and specificity as it appears to be a robust tool for mycobacteria recognition in selected clinical situations, as well as for quantitation in experimental settings [37], [45], [47]–[50].

One of the first studies based on PCR was carried out in 1990 by Williams and colleagues They established a procedure for detecting *M. leprae* DNA in infected tissues [51]. The PCR test was specific and detected *M. leprae* DNA in biopsies from leprosy patients. The evolution of PCR, as evaluated by technical issues (time and handling) but also by molecular and clinical sensitivity, is remarkable. In the early 1990s, radioactive probes were required to increase PCR sensitivity, and, hence, to overcome problems inherent to radioactivity, nonradioactive probes were developed [43]. Also, nested PCR was introduced and employed to increase specificity and sensitivity, to avoid the use of radioactive probes, and to shorten the time required to obtain a result [44]. Both studies demonstrated the emerging potential of PCR technology in the rapid detection and definitive identification of small numbers of *M. leprae* in clinical specimens.

The quality and the quantity of the isolated nucleic acid as well as the PCR target product size had tremendous effects on the success of amplification methods. Therefore, several protocols have been described for purification and amplification of *M. leprae* DNA, RNA, or both. Extractions that do not involve any purification step, for example, can inhibit the polymerase reaction due to impurities in the extract, as described by de Wit in 1991 [52]. Nevertheless, methods employing commercial kits have been consistently used and seem to be effective [53], [54], although conditions to evaluate repeatability and other parameters to further explore the potential of the technique are still lacking. In parallel, extraction methods proved to be suitable for formalin-fixed samples and further amplification under certain conditions [55]. Samples can also be easily stored in 70% ethanol and FTA cards for *M. leprae* DNA detection [56] exhibiting similar recovery rates.

Furthermore, the size of the PCR fragment amplified has to be taken into account as adaptation of conventional [52], [57] to real-time PCR assays [47] requires shorter length amplicons. An overall assessment of the impact of the PCR technology in leprosy diagnosis can be observed in Table 1 using skin biopsy samples as an example. Also, irrespective of whether the detection method used is conventional or real-time PCR, smaller PCR products allow for better amplification efficiency from DNA extracted from either formalin- or ethanol-fixed or fresh tissues. In fact, an important advance has been the real-time PCR technology. This method allows direct quantitation of bacterial DNA content in clinical samples and has improved turnaround time and cost effectiveness (Table 1), permitting more reliable results. The procedure follows the general principle of PCR, and its key feature is that the amplified DNA or cDNA (complementary DNA) is quantitated as it accumulates in the reaction in real time after each amplification cycle. These real-time methods have improved slightly but consistently the analytical and clinical sensitivity when PB patients' samples were assessed in skin samples [37], [48]. In addition, analyses using real-time PCR showed that total DNA content estimated by molecular levels could be correlated to bacterial load, corroborating the clinical data, which can be useful to determine a molecular bacteriological index, helping to define the clinical form of patients [37], [48], [50]. Nevertheless, while PCR diagnosis is not needed for lepromatous patients with high bacillary load and high number of lesions, it is extremely helpful for the diagnosis of the already-mentioned situations such as clinical presentations with scarce number of *M. leprae* bacilli and difficult-to-diagnose patients.

**Table 1:** Selected results obtained by PCR assays tested in frozen and fresh skin biopsies from leprosy patients.

DNA Targets	PCR Method	Results	References
Proline-rich antigen (pra-36 KDa)	PCR-Southern hybridization	87–100% MB, 36–60% PB	[42], [52], [96]
	TaqMan real-time PCR	89% BI^+^, 33% BI^−^.	[47]
18 kDa	PCR-Southern hybridization	99% MB, 74% PB.	[97]
		The specificity was 100%, and sensitivity ranged from 50% to 83%. A group of patients with other skin disorders was also tested.	[43]
RLEP	PCR-Southern hybridization	100% BI^+^ or BI^−^.	[35]
	PCR	100% MB, 73%, PB.	[57], [98]
RLEP and TTC repeat	Multiplex-PCR	100% MB, 83% PB.	[99]
RLEP	TaqMan real-time PCR	The specificity was 73%, and sensitivity was 91%.	[63]
Ag85B	TaqMan real- time PCR	100% MB, 80% PB.	[37]
		The specificity was 100%, and sensitivity was 56%.	[63]
16S	Taqman real-time PCR	The specificity was 100%, and sensitivity was 51%.	[63]
16S	SyBr green real-time PCR	100% MB, 50% PB.	[48]

## PCR for Diagnosis of Difficult Cases

### Pure neural cases

PCR can aid in defining leprosy diagnosis in suspected patients with clinically suggestive or atypical lesions presenting with negative baciloscopy and inconclusive histopathology. This is true for primary neuritic or PNL patients, who are easily missed and misdirected since they do not exhibit cutaneous lesions [11]. Timely treatment is imperative in these cases because, once nerve fibrosis occurs, damage is permanent and irreversible. Ridley and Jopling (R&J) postulated that PNL might occur across the spectrum from borderline lepromatous (BL) to tuberculoid (TT) forms [58], but, in our experience, the PNL cases are indeterminate or borderline tuberculoid (BT) [59]. In fact, these patients cannot be classified according to the R&J system because of the absence of skin lesions and clear histopathological features in the nerve. Nevertheless, a general WHO classification (paucibacillary) is used as none of them present bacilli in the slit-skin smears. A careful investigation examining skin biopsies (areas of skin hypoesthesia) described the histopathological features in the cutaneous lesions of PNL cases [59]. The assessment of PNL skin biopsies showed histopathological features consistent with normal skin, although indeterminate or borderline tuberculoid histological alterations were also detected. However, analysis of patients' nerve biopsies often showed detectable bacilli using Wade staining. It is curious that, even in endemic countries, leprosy is assumed to be a dermatologic disorder. Therefore, it is quite challenging to diagnose PNL cases [10]. Neurologists are not expecting leprosy as a probable cause of peripheral neuropathy and, thus, laboratory techniques (i.e., histological evaluation, PCR from biopsy, and/or PGL-I in the serum) may be used along with pertinent clinical and electroneuromyographical data [12]. In clinical practice, PCR is very useful in detecting *M. leprae* DNA in nerve specimens that have been shown to be bacteriologically negative by other methods of detection. In fact, Jardim and coworkers [12] demonstrated that *M. leprae* infection in PNL cases is diagnosed most often by PCR, followed by anti-PGL-I antibodies and direct observation of the bacteria (acid-fast bacilli [AFB]). Hence, PCR is helpful and is being used as a confirmatory and diagnostic routine tool in difficult-to-diagnose cases such as PNL [60]–[62].

### Differential diagnosis to other conditions

In an endemic country, leprosy is suspected in patients with anaesthesic lesions, although not necessarily so. PCR could be of immense help for dermatological differential diagnosis in hypochromic or granulomatous lesions, such as pityriasis alba, leishmaniasis, cutaneous tuberculosis (TB), and sarcoidosis, among other skin diseases in which pathological examination is inconclusive. There are few papers evaluating the application of PCR to solve this matter. Our retrospective analysis testing different gene targets (Ag 85B [37], *sodA* and 16S rRNA [45], and repetitive sequence [RLEP] [50]) using a panel of samples from patients previously diagnosed by pathologists and dermatologists, provided interesting information [63]. When we include a higher proportion of paucibacillary samples (single skin indeterminate and tuberculoid forms), rates of PCR positivity decrease, but we were able to ascertain 50% of sensitivity. Obviously, that is expected since leprosy diagnosis is challenging in exactly these situations. Also, a group of other dermatological diseases were included as a negative control group, and the results suggest [63] that some positive samples for PCR were misdiagnosed. These samples were defined initially as other dermatological diseases, but patients developed leprosy 10 years later, suggesting that PCR for *M. leprae* DNA could be a very early detection test for leprosy [63].

### PCR for treatment monitoring

In 1991, de Wit and coworkers [52] validated a PCR assay based on the selective amplification of a 530-bp fragment of the gene encoding the proline-rich antigen of *M. leprae* using clinical samples. They were able to detect the presence of *M. leprae* DNA on frozen biopsy sections from all untreated AFB-positive patients and 56% of the treated AFB-negative patients. The authors believed that PCR positivity reflected the presence of viable bacilli at the time of biopsy since a strong host immune response could result in killing of *M. leprae* and breakdown or clearance of its DNA in negative PCR samples.

Subsequent studies confirmed that PCR technology could be useful both for diagnosis and for assessment of viable load, as a reduction in signals was found to correlate with loss of viability. A follow-up study using patient's biopsies confirmed that *M. leprae* is rapidly killed after one month of multidrug therapy (MDT) treatment since MB cases declined by 54.3% and PB cases by 61.8% of initial positivity rate [42]. However, because of the persistence of weak signals, in some cases a long time after effective treatment [64], [65], the authors concluded that DNA-based PCR assays lack the sensitivity to estimate any real impact of treatment on bacterial viability. Similarly, in 2001, Santos and colleagues [66] tested a PCR assay on different samples of leprosy patients that had completed MDT treatment. This PCR assay targeted a RLEP described previously and was able to detect *M. leprae* from hair bulb, blood, nasal secretion, lymph, and skin biopsy samples. Results demonstrated that 54.5% of the individuals were PCR positive in at least one of the samples 8 years after completion of MDT. However, it was not possible to draw final conclusions on the clinical significance of PCR positivity since assays were based on DNA detection and did not reflect viable bacilli.

To overcome this problem, several studies were conducted using reverse transcriptase-PCR (RT-PCR)-based assays for *M. leprae* viability estimation. It has been noted that an RNA-based test is likely to reflect only nucleic acids from living organisms, as the turnover rate of RNA is high, particularly in prokaryotes. Hence, methods based on a quantitative estimation of RNA levels in the tissues have been useful for monitoring therapeutic responses [67]–[69]. A PCR assay for monitoring bacterial clearance in leprosy patients during chemotherapy based on *M. leprae* 16S rRNA gene expression was described [67]. After 6 months of MDT, they found that 44% of MB patients and 4% of PB patients tested still showed viable bacilli.

However, this assay was based only on the 16S rRNA, a relatively stable RNA species under several conditions, and was unable to detect rapid killing of *M. leprae*. Also, given that 16S rRNA gene is a housekeeping gene, a major drawback of these previous works is the lack of a gene target to normalize the template as an indicator of bacterial numbers in the specimen. Thus, Martinez and coworkers [45] propose a real-time PCR integrated approach based on RNA/DNA ratios for viability determination, i.e., decrease of *M. leprae*-specific RNA is evaluated as a function of total *M. leprae* DNA content. Previous results demonstrated that a significant decrease in viability could be seen in vitro in as little as 48 hours post-treatment with rifampin. Also, analysis of human biopsies confirmed the correlation of MDT treatment and decline of gene expression level [45]. This new approach may be helpful in the follow-up of leprosy patients on treatment and determination of drug resistance [70]. Also, other researchers have been using the same approach to estimate the viability in *M. ulcerans* (Buruli ulcers) and also in pathogenic fungi [71], [72]. Interestingly, this method has been applied to estimate *M. leprae* viability in in vitro assays [73], [74].

A recent and similar approach to monitor the effectiveness of chemotherapy using hsp18 as the gene target was developed by Lini and coworkers [75]. The copy number of bacterial DNA and hsp18 mRNA was estimated from 47 leprosy patients during treatment using paraffin-embedded biopsy samples. A reduction in DNA and mRNA during chemotherapy was observed, and hsp18 mRNA could not be detected in patients who underwent 2 years of MDT. Ten years ago, WHO recommended shortening the treatment to 12 months, although no molecular studies compared both regimens. Anyways, since there are no clear epidemiological alterations in relapse rates as examples, it could be suggested that indeed all *M. leprae* is being killed after 12 months of treatment, although a considerable amount of *M. leprae* DNA remained in the skin after 2 years of MDT. Also, recent molecular epidemiological evidence indicates that reinfection is more common than relapse in second episodes of the disease emerging [76].

### PCR for the study of leprosy transmission and household contact surveillance

It is clear that household contact examination and follow-up is a determinant of leprosy control [3], [4], [77]. The arsenal of laboratory exams to screen this population could increase detection and early diagnosis. Several findings about leprosy indicate that *M. leprae* transmission mainly occurs by airborne droplet inhalation of *M. leprae*. Therefore, for purposes of clinical practice, the application of PCR for detection of *M. leprae* DNA in nasal swab samples from healthy individuals and household contacts has been reported [36], [39], [78], [79]. Results provided evidence that a majority of MB patients carry *M. leprae* in their nasal mucosa and that carriage of *M. leprae* occurs among healthy people living in an area where leprosy is endemic [78]–[80]. In household contacts, detection of *M. leprae* DNA by PCR in nasal swabs does not infer whether the contact will progress to active disease. DNA detection rates in nasal swabs in contacts vary from 1 to 10% (Table 2), which sometimes depends on the clinical form of the index cases. The data are not conclusive because prospective studies enrolling high numbers of contacts are still lacking. However, the high positivity rates observed among healthy individuals (Table 2) question the feasibility of the use of PCR in this site to predict the risk of developing the disease. Nevertheless, it has been shown that positive PGL-I test among contacts can increase the risk of developing leprosy [81], [82]. More recently, a very interesting study indicates that, indeed, the risk of progressing to active disease increases if a contact tests positive for PCR in the blood [83]. Thus, it is likely that a PGL-I test in combination with PCR could help identify the population at highest risk among household contacts [84].

**Table 2:** Selected data showing PCR assays tested in nasal swabs or blood from healthy individuals and household contacts.

DNA Targets	PCR Method	Sample	Population	Results	References
Proline-rich antigen (pra-36 KDa)	PCR-Southern hybridization	Nasal swab	Healthy	7.8%	[80]
	PCR-ELISA	Nasal swab	Healthy	7.8%	[79]
RLEP	PCR	Nasal swab	Healthy	31%	[78]
		Nasal swab	Household contacts	5.2% MB IC*, 3.8% PB IC.	[84]
		Nasal swab	Household contacts	10% MB IC, 6% PB IC.	[100]
RLEP and TTC repeat	Multiplex-PCR	Nasal swabs	Household contacts	11% MB IC, 1.3% PB IC.	[99]
ML0024	Real-time PCR	Blood	Household contacts	1.2%	[83]
RLEP	Nested PCR	Blood	Household contacts	6.25%	[53]

*household contacts with multibacillary patients as index case (IC).

It is believed that humans are the only significant reservoir of infection in leprosy, but recent investigations reported the presence of *M. leprae* DNA in wild armadillos and environmental samples. Thus, studies in areas of high prevalence of the disease confirmed the presence of *M. leprae* in water samples in Indonesia [85] and soil in India [86] as potential sources of continued transmission of the disease. Also, Job and coworkers suggested that skin and nasal epithelia of untreated MB leprosy patients contribute to the shedding of *M. leprae* into the environment, which in turn increases the risk for household contacts [87]. In addition, Truman and collaborators [2] used whole-genome sequencing to show that wild armadillos and American patients with leprosy in the southern United States are infected with the same strain of *M. leprae*. They were able to confirm that about a third of the leprosy autochthonous cases that arise each year in the United States almost certainly result from contact with infected armadillos.

## Technical Limitations and Future Perspectives of PCR-Mediated Leprosy Diagnosis

Although PCR could be a useful tool for the detection of subclinical infection, only a few investigations have consistently associated the presence of the *M. leprae* DNA with further development of the disease among household contacts [83]. However, PCR results associated with a serological test could improve the predictive value of PCR technology in leprosy diagnosis. In addition, the PCR-integrated approach based on RNA/DNA ratios for viability determination can be useful for assessment of infection rate with *M. leprae* within a population in the future. Earlier diagnosis of leprosy will be of great value in preventing more severe disease that may lead to disabilities. Chemotherapy at an early stage could preclude leprosy transmission and the consequences of late diagnosis.

In clinical practice, the detection of *M. leprae* by PCR in patients with negative baciloscopy or inconclusive histopathology would be of great value to define leprosy diagnosis. Thus, choosing the right target for an improvement in sensitivity is important. The use of a repetitive sequence as a PCR target DNA, for example, provides the advantage of higher sensitivity over other targets in the DNA because it is present at multiple sites in genomic DNA [88]. However, specificity of a repetitive sequence as a PCR target is an issue since we observed that it is lower than other assays. For this reason, although it seems encouraging, highest sensitivity has to be interpreted with great care. The RLEP gene target is highly conserved and, as a result, many homologous sequences may be present in other *Mycobacterium* species that have not been thoroughly investigated, generating false positive results, as reported for the *M. tuberculosis* IS6110 marker elsewhere [89]. So far, gene targets such as 16S and Ag85B could be considered a good cost-benefit ratio concerning specificity and sensitivity (Table 1) [63]. This also argues against results detecting “*M. leprae*” DNA in water or soil [85], [86].

For routine application of PCR, some operational aspects such as the invasive nature of the sample collection should be considered. Therefore, comparative studies of different types of clinical samples for leprosy diagnosis have been carried out. Less invasive samples such as blood, urine, nasal swab, hair bulbs, and, most importantly, slit-skin smears were accessed, and, although results were encouraging, they were less efficient than those obtained with skin biopsies; skin biopsies would be the best sample for household contacts screen if not for the ethical considerations [35], [90]. Nevertheless, amplification of *M. leprae* in blood samples, for example, gives inferior results in comparison to those using other types of clinical material [35]. Even though biopsy sampling of the lesion is obtained through an invasive method, it is the choice in most studies as it provides the highest PCR positivity rates.

So far, a well-characterized, commercial test for detection of *M. leprae* DNA in patients' samples is not available. Therefore, many labs continue to report results using their own definitions of sensitivity and specificity, and, in most cases, the results are not comparable across different clinical applications. Currently, several specific *M. leprae* genes of interest have been identified, and, thus, assays based on existing simple automated machines such as the GeneXpert assay for diagnosis of *M. tuberculosis* infection [91] could be developed for leprosy. The Xpert MTB/RIF detects DNA sequences specific for *M. tuberculosis* and rifampicin resistance by PCR and is a major advance for TB diagnostics, especially for multidrug-resistant (MDR) TB and HIV-associated TB. Additional new technologies for miniature “lab on a chip” [92] and lateral flow assays [93]–[95] are also progressing so quickly that such assays would be feasible at point–of-care to improve clinical management decisions on leprosy diagnosis.

No data exist concerning the relative performance of different laboratories and methods for *M. leprae* DNA detection. An external quality assurance study on diagnostic proficiency that includes certifying and publishing the results in a comparative and anonymous manner would be highly recommended for leprosy diagnosis. Validation of paramount issues like adequate clinical material, nucleic acid extraction methods, sensitivity, specificity, PCR inhibition, and control of contamination will assure a reliable diagnosis of the disease. Thus, comparative testing of characterized samples would be a direct way to identify weaknesses of individual laboratories or certain methods. Furthermore, the positive predictive value (PPV) is another means of evaluating the usefulness of a diagnostic test as it reveals the probability that a positive result reflects the underlying condition being tested for. Its value does, however, depend on the prevalence of the disease, which may vary. Similarly, the negative predictive value (NPV) determines the proportion of patients with negative results who are correctly diagnosed. Although very useful, these values are difficult to apply to leprosy diagnosis due to the lack of a true gold standard method.

## Conclusions

Overall, extensive evaluation of PCR tests in field studies has shown that DNA-based PCR assays can be 100% specific, while the sensitivity ranges from 34 to 80% in patients with PB forms to greater than 90% in patients with MB forms of the disease (Table 1). Also, since finding *M. leprae* is crucial in the confirmatory diagnosis of early leprosy, the use of PCR technique to enhance the ascertainment of difficult cases such as early PB and PNL is advisable and important in reaching a definitive diagnosis (Box 2). Thus, performing PCR to detect *M. leprae* DNA in difficult-to-diagnose cases can be executed in thousands of samples, favoring early identification and early treatment and helping to interrupt the transmission chain. Moreover, definitions of *M. leprae* strains could be very helpful in leprosy transmission. Undoubtedly, there is a future for PCR-based methods in relation to leprosy since these methods provide options for confirmation of diagnosis, treatment follow-up, detection of resistance, and, especially, support for the diagnosis of difficult cases such as PNL and PB.

**Box 2. Top Five Papers in the Field**

1. Reis EM, Araujo S, Lobato J, Neves AF, Costa AV, et al. (2013) Mycobacterium leprae DNA in peripheral blood may indicate a bacilli migration route and high-risk for leprosy onset. Clin Microbiol Infect. E-pub ahead of print. doi: 10.1111/1469-0691.12349
2. Ezenduka C, Post E, John S, Suraj A, Namadi A, et al. (2012) Cost-effectiveness analysis of three leprosy case detection methods in Northern Nigeria. PLOS Negl Trop Dis 6: e1818.
3. Martinez AN, Ribeiro-Alves M, Sarno EN, Moraes MO (2011) Evaluation of qPCR-based assays for leprosy diagnosis directly in clinical specimens. PLOS Negl Trop Dis 5: e1354.
4. Truman RW, Andrews PK, Robbins NY, Adams LB, Krahenbuhl JL, et al. (2008) Enumeration of Mycobacterium leprae using real-time PCR. PLOS Negl Trop Dis 2: e328.
5. Jardim MR, Antunes SL, Santos AR, Nascimento OJ, Nery JA, et al. (2003) Criteria for diagnosis of pure neural leprosy. J Neurol 250: 806–809.
